# RNA sequencing and proteomic profiling reveal different alterations by dietary methylmercury in the hippocampal transcriptome and proteome in BALB/c mice

**DOI:** 10.1093/mtomcs/mfab022

**Published:** 2021-04-23

**Authors:** Ragnhild Marie Mellingen, Lene Secher Myrmel, Kai Kristoffer Lie, Josef Daniel Rasinger, Lise Madsen, Ole Jakob Nøstbakken

**Affiliations:** Institute of Marine Research, Bergen, Norway; Department of Biomedicine, University of Bergen, Bergen, Norway; Institute of Marine Research, Bergen, Norway; Institute of Marine Research, Bergen, Norway; Institute of Marine Research, Bergen, Norway; Institute of Marine Research, Bergen, Norway; Department of Biology, University of Copenhagen, København, Denmark; Institute of Marine Research, Bergen, Norway

**Keywords:** methylmercury, RNA sequencing, mice, proteomic, hippocampus

## Abstract

Methylmercury (MeHg) is a highly neurotoxic form of mercury (Hg) present in seafood. Here, we recorded and compared proteomic and transcriptomic changes in hippocampus of male BALB/c mice exposed to two doses of MeHg. Mice were fed diets spiked with 0.28 mg MeHg kg^–1^, 5 mg MeHg kg^–1^, or an unspiked control diet for 77 days. Total mercury content was significantly (*P* < 0.05) increased in brain tissue of both MeHg-exposed groups (18 ± 2 mg Hg kg^–1^ and 0.56 ± 0.06 mg Hg kg^–1^). Hippocampal protein and ribonucleic acid (RNA) expression levels were significantly altered both in tissues from mice receiving a low dose MeHg (20 proteins/294 RNA transcripts) and a high dose MeHg (61 proteins/876 RNA transcripts). The majority but not all the differentially expressed features in hippocampus were dose dependent. The combined use of transcriptomic and proteomic profiling data provided insight on the influence of MeHg on neurotoxicity, energy metabolism, and oxidative stress through several regulated features and pathways, including RXR function and superoxide radical degradation.

## Graphical Abstract

**Graphical Abstract:**
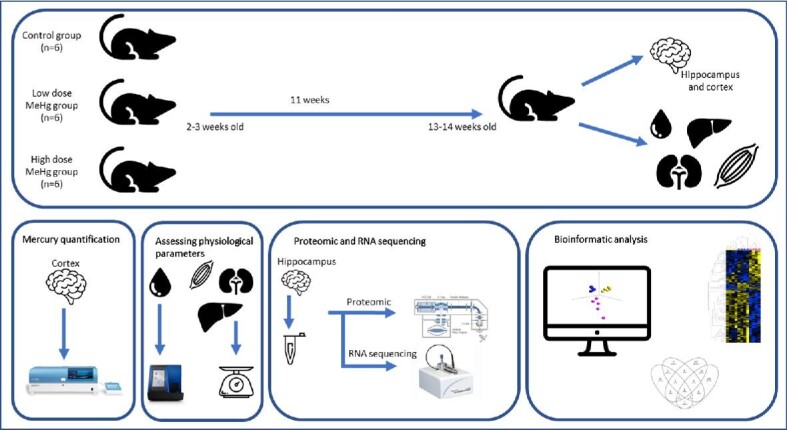
Juvenile mice, exposed to low (0.04 mg kg^–1^ bw^–1^ day^–1^) and high dose (0.67 mg kg^–1^ bw^–1^ day^–1^) MeHg, revealed differential expression of brain proteome and transcriptome.

## Introduction

The organic methylmercury (MeHg) originates from both natural and anthropogenic sources and is abundantly spread in the atmosphere and biosphere.^1–^^4^ The compound biomagnifies along the aquatic food chain,^5^ and the main exposure route of MeHg for humans is through seafood consumption.^6^ However, mercury (Hg) exposure differs highly among consumers, as different types of seafood with varying levels of MeHg are consumed with different frequency among populations.^7–^^11^ Extensive studies have been conducted based on major contamination incidents in Japan and Iraq where levels of exposure to MeHg were high^12–^^17^; however, the levels of MeHg in most consumed commercial fish species are often relatively low.^11^ Further, MeHg exposure exerts a non-monotonic dose response rather than a linear dose response.^18,19^ Thus, effects reported at low exposure doses in animal trials are not necessarily observed at high doses of MeHg exposure.^18,19^ The European Food Safety Authority (EFSA) did in their latest evaluation of MeHg establish a tolerable weekly intake level for MeHg at 1.3 µg kg^–1^ bw (EFSA 2012). However, since recent studies discuss the validity of such a threshold dose, research on low dose effect of MeHg is required.

Factors determining the grade of mercury toxicity comprise the quantity of the metal ingested, exposure time, and the chemical form of mercury.^3,20,21^ In addition to a long half-life of MeHg, age and habitat are determinants affecting the levels of accumulated mercury in the brain of both humans and wildlife animals.^22–^^26^ As different types of seafood contain different levels of MeHg and the intake of seafood differs, the levels of Hg exposure in humans will also vary. A comprehensive understanding of MeHg toxicity at a range of doses is therefore important. MeHg is one of the most toxic forms of mercury able to cross the blood–brain barrier and influence the neurological system, causing the developing and maturing brain to be particularly vulnerable.^2,27^ Effects of MeHg on behavioral performance in adolescent mice have been observed,^19^ and whereas some effects of MeHg exposure are well documented, the underlying mechanisms are still not fully uncovered. The hippocampus is associated with learning, memory, and IQ, factors pertinent in MeHg toxicity, and is therefore an important target region of the brain for effects of MeHg.^87^

During the last decade, proteomic tools have been increasingly used to identify novel processes and biological pathways regulating toxicological responses to different substances,^28^ such as the elucidation of mechanisms underlying MeHg neurotoxicity in experimental animal model systems.^29–^^37^ Both proteomic^38^ and transcriptomic^39^ tools have been extensively used to investigate MeHg toxicity in different species. However, to date only a few have used an integrated application of proteomic and transcriptomic data to unravel mechanisms underlying MeHg neurotoxicity.^40^ By combining different omics techniques, additional cellular and molecular mechanisms may be identified and thus, more molecular changes at low exposure levels may be detected.^41^ This was demonstrated when the neurotoxic potential of persistent organic pollutants was investigated in juvenile female BALB/c mice, by the combined use of genes and proteins in a targeted biological network analyses.^42^ Knowledge gaps still exist for a full understanding concerning the cellular processes and downstream biochemical pathways governing MeHg toxicity.^43^ Hence, combining transcriptomic and proteomic approaches followed by pathway analysis software may uncover molecular mediators both at the transcript and protein levels, providing additional evidence for possible mechanisms of action underlying the observed MeHg toxicity.^41,44^ In our study, we implemented a sensitive approach combining proteomics and ribonucleic acid (RNA) sequencing to elucidate the difference between the sub-chronic exposure of a high dose of 5 mg MeHg kg^–1^ feed or a more environmentally relevant low dose of 0.28 mg MeHg kg^–1^ feed in BALB/c mice during adolescence and early adulthood.

## Materials and methods

### Experimental animals and study design

Male mice of the inbred BALB/c strain were obtained from Taconic Biosciences (Ejby, Denmark) at the age of 2–3 weeks weighing 10 ± 2 g (SD). Young mice were chosen due to the increased sensitivity to neurotoxic substances during adolescence, a developmental period where important alterations and maturation of the brain occur.^45,46^ The mice were individually housed (NexGen IVC, Allentown Inc., Allentown, NJ, USA) and kept in a controlled environment at 24 ± 2°C, 50% humidity, and a 12/12 h light/dark cycle. The mice were given sufficient feed to ensure growth and had *ad libitum* access to water.

After 5 days of acclimatization, mice were weighed and assigned to experimental groups (*n*/group = 6), ensuring a similar mean body mass, and fed experimental diets spiked with MeHg or an unspiked control diet for 11 weeks. The mice were weighed once a week and fed three times a week with experimental diets. Body composition was assessed at the end of the experiment using a Minispec LF50mq7.5 NMR Analyzer (Bruker Corporation, MA, USA), and percentage body fat and lean mass were calculated. The animal trial and associated experimental protocols were approved by the Norwegian Food Safety Authority (Mattilsynet; FOTS ID: 12400).

### Experimental diets

The AIN-93G purified diet (Harlan Laboratories Ltd, Indianapolis, IN, USA) served as a basis for all experimental diets. All ingredients required for preparation of the diets were mixed in a Crypto Peerless blender, EF20 (Crypto Peerless, Halifax, UK) and finalized feeds were stored at −20°C. The experimental diets were spiked with 0.28 mg MeHg kg^–1^ and 5 MeHg mg kg^–1^. An approximation of exposure doses giving a rough estimate of MeHg exposure per bodyweight was conducted. The estimate was based on total feed intake, the corresponding MeHg content in feed, divided by length of experiment (in days) and average body weight throughout the study, resulting in the approximate exposure doses of 0.04 mg MeHg kg^–1^ bw^–1^ day^–1^ (low dose; LD) and 0.67 mg per kg^–1^ bw^–1^ day^–1^ (high dose; HD).

In brief, for the preparation of diets, a MeHg stock was made, with a concentration of 1 mg MeHg ml^–1^, where 116.4 mg MeHg-Cl [methylmercury(II)chloride, Sigma-Aldrich, Darmstadt, Germany] was mixed with 1 ml ethanol and added to 49 ml dH_2_O. Further, 56.4 mg cysteine (L-cysteine from non-animal source; Sigma-Aldrich, Darmstadt, Germany) was mixed with 50 ml dH_2_O and added to the MeHg solution. The molar ratio MeHg:Cys was 1:1. The HD stock was diluted with dH_2_O to prepare the LD stock with a concentration of 0.058 mg MeHg ml^–1^. A cysteine stock was made by mixing 56.4 mg cysteine with 99 ml dH_2_O and 1 ml ethanol, and the stock was added to the control (Ctr) feed.

The added levels of Hg were verified by inductively coupled plasma mass spectrometry (ICP-MS) analysis (described in Section 2.4). The Hg levels in the Ctr feed were below the limit of quantification (LOQ) for the instrument (*n* = 4) and the LD and HD MeHg levels in the experimental diets were in accordance with nominal concentrations within the uncertainty range (±20%) of the method (*n* = 4).

### Tissue and feces sampling

At termination, the mice were anesthetized with isoflouran (4%) using an Univentor 400 Anesthesia Unit apparatus (Univentor Limited, Zejtun, Malta) with airflow at 404 ml min^–1^. The mice were euthanized through bilateral thoracotomy and blood sampling through cardiac puncture. Hippocampus, cortex, tibialis, quadriceps femoris, kidneys, and liver were dissected out, weighed, and collected in plastic bags. All organs were snap-frozen in liquid nitrogen and further stored at −80°C.

### Mercury quantification

Total Hg (THg) was determined in cortex by direct mercury analysis (DMA-80, Milestone, Sorisole, Italy) as described by the United States Environmental Protection Agency.^47^ Two certified reference materials were used: dogfish liver (Dolt-4; National Research Council Canada, Ottawa, Ontario, Canada) and tuna fish (ERM-CE464; European Reference Material ERM). The obtained values of the reference material were within the uncertainty of the method (±20%). LOQ of the method is 0.08 ng Hg.

THg concentrations in diets were quantified by ICP-MS (Thermo iCAP Q, ThermoFisher Scientific, Waltham, MA, USA) as described by Julshamn *et.al*.^48^ The apparatus was equipped with a FAST SC-4 DX auto sampler (Elemental Scientific, Omaha, NE, USA). The samples were decomposed by UltraWAVE, Single Reaction Chamber Microwave Digestion System (Milestone, Sorisole, Italy) prior to the analyses. Rhodium, germanium, and thulium were used as internal standards to correct for any drift of the instrument. Two certified reference materials were used: lobster hepatopancreas (Tort-3; National Research Council Canada, Ottawa, Ontario, Canada) and oyster tissue (SRM-1566b; National Institute of Standards and Technology, Gaithersburg, MD, USA). The obtained values of the reference material were within the uncertainty of the method (±20%). The LOQ of this method is 0.005 mg kg^–1^ for mercury.

### Proteomic analysis

A total of 12 hippocampus samples (four mice per exposure group) were prepared for proteomic analysis. Sample preparation and protein mass spectrometry were performed as previously described,^49^ following standard protocols and procedures at the Proteomics Unit at the University of Bergen, Norway (PROBE). In short, proteins were extracted and solubilized in lysis buffer (4% SDS, 0.1 M Tris–HCl, pH 7.6). Samples were subjected to sonication (Q55 Sonicator, Qsonica, CT, USA), centrifuged (10 min at 13 000 rpm), and supernatants were collected. Protein concentration was determined (Pierce™ BCA Protein assay kit, Thermo Scientific) and samples were digested with trypsin following a filter-aided sample preparation digestion protocol as described by Wiśniewski *et al.*^50^ Tryptic peptides (0.5–1 µg) dissolved in 2% acetonitrile and 0.1% formic acid were injected into an Ultimate 3000 RSLC system (Thermo Scientific, CA, USA) connected to a linear quadrupole ion trap-orbitrap (LTQ-Orbitrap Elite) mass spectrometer (Thermo Scientific, Bremen, Germany) equipped with a nanospray Flex ion source (Thermo Scientific). Raw data obtained in data-dependent acquisition mode were analyzed as described by Tyanova *et al.*^51^ In short, MaxQuant^52^ with the built-in search engine Andromeda^53^ was used for protein identification and protein quantification. MaxQuant (version 1.6.4.0) parameter settings were set as described before.^49^ Only reviewed protein sequences of the mouse proteome (UniProt proteome: UP000000589; accession date: 08.11.2018) were used for protein identification. False discovery rates for peptide and protein identification were set to 1%; only unique peptides were used for label-free quantification (LFQ). MaxQuant data were processed further using Perseus (version 1.6.10) as described in Tyanova *et al.*^54^ Protein expression data including LFQ intensities, statistical significance, fold changes, and protein identification features including accession numbers, protein names, isoelectric point, molecular weight, and protein identification metrics are provided in Table S1.

### RNA sequencing

Five hippocampus samples from each group were included for RNA sequencing. Total RNA was extracted from each sample using BioRobot^®^ EZ1 and RNA Tissue Mini Kit (Qiagen, Hilden, Germany), including DNAase treatment as instructed in the RNA Tissue Mini Kit manual (Qiagen, Hilden, Germany). RNA quality was analyzed using a NanoDrop ND-1000 UV–vis Spectrophotometer (NanoDrop Technologies, Wilmington, USA). Agilent 2100 Bioanalyzer and RNA 6000 Nano LabChip kit (Agilent Technologies, Palo Alto, USA) were used to validate RNA integrity. Samples had 260/280 and 260/230 ratios between 2.0 and 2.1 and between 2.0 and 2.2, respectively. The average RNA integrity number of all samples was 8.3 ± 0.4. Sequencing and library preparation were performed by the Norwegian Sequencing Centre (www.sequencing.uio.no). DNA libraries were prepared using 90 ng total RNA input to the TruSeq Stranded RNA Library Prep Kit (Illumina) and standard Illumina adaptors for multiplexing. Libraries for each individual sample were sequenced using the NextSeq Illumina platform according to the manufacturer’s instructions, generating single-end 75 bp read libraries with an average library size of 15 ± 2 million reads. TrimGalore 0.4.2 tool (https://github.com/FelixKrueger/TrimGalore) was applied for removing adaptors and for quality trimming using default parameters. Sequence quality for each sample was investigated using FastQC imbedded in TrimGalore. Libraries were mapped individually to the *Mus Musculus* genome (Ensembl genome build NCBIM37, downloaded July 2015) using the Hisat2 short read aligner version 2.0.4. Transcript abundance for the individual libraries was estimated using FeatureCounts^55^ of the Subread package (http://subread.sourceforge.net/). Count data was normalized using the DESeq2 (version 1.18.1)^56^ included in the Bioconductor R package (version 3.4.4). Features for which the row sum was <10 reads were excluded from further analysis prior to normalization and differential expression analysis. RNA expression data, including gene identification, statistical significance, and fold changes, are provided in Table S2, while all raw data have been uploaded to the gene expression omnibus (GEO) with accession number GSE135381.

### Statistical analyses and bioinformatics

Tissue levels of mercury and physiological parameters were statistically evaluated using GraphPad Prism^®^ 7.05 (GraphPad Software Inc., La Jolla, CA, USA) using one-way analysis of variance (ANOVA) followed by Tukey's multiple comparison test. Normality was tested by D'Agostino and Pearson normality test and Shapiro–Wilk normality test, and homogeneity of variance was assessed using Brown–Forsythe and Bartlett's test. If these assumptions were not met, statistics were performed on Box-Cox transformed data.

Qlucore Omics Explorer 3.5 (Qlucore AB, Lund, Sweden) was used for statistical analysis of proteomic and transcriptomic data. Prior to statistical analysis, pre-processed RNA seq data were log2 transformed; proteomics data were analyzed as output by Perseus using log10 transformed LFQ intensity data. Data were analyzed using ANOVA comparing the three groups followed by planned contrasts, to investigate the specific comparisons between groups. In the omics analyses, *P*-values were used at a threshold of *P* < 0.05 for statistical significance. The use of *P*-values has previously been recommended to increase the sensitivity of omics analyses^57^; however, this may also increase the chance of obtaining false positives of which the reader should be aware. Therefore, multiple test-corrected *q*-values are also reported in Tables S1 and S2. The use of *P*-values was chosen since the simultaneous application of RNA sequencing and proteomics reduces the chance of obtaining false positives by increasing the weight of evidence. The data were further examined by principal component analysis (PCA). Nomenclature of proteins and RNA transcripts are based on the UniProt database and are denoted in the text with uppercase and lower case letters, respectively. Overlapping features between proteins and RNA transcripts are denoted with italic uppercase letters.

Biological network analyses were conducted in the software Ingenuity Pathway Analysis (IPA; Qiagen, Redwood City, CA, USA). The entire datasets of proteins and transcripts were imported to IPA using UniProt and Ensembl Genomes accession numbers for proteins and transcripts, respectively. A threshold of *P* < 0.05 was chosen for accepting statistical significance; human, rats, and mice were the species of selection; and the settings for specification of tissues and cells were narrowed down to “nervous system,” “central nervous system (CNS) cell lines,” and “neuroblastoma cells.” “Core analysis” (using default settings) was performed on proteins and RNA transcripts separately in each group, for further manual inspection and comparison. Comparison analyses were done in IPA to summarize similarities between LD and HD, as well as overlap between RNA seq data and proteomic data. For illustrative purposes, the online software Venny 2.1.0^58^ was used to generate all Venn diagrams.

## Results

To explore how dietary MeHg influences protein and RNA expression in hippocampus, we sub-chronically exposed BALB/c mice to a low dose MeHg (LD; 0.28 mg Hg kg^–1^ feed, corresponding to ∼0.04 mg MeHg kg^–1^ bw^–1^ day^–1^), a high dose MeHg (HD; 5 mg Hg kg^–1^ feed corresponding to ∼0.67 mg MeHg kg^–1^ bw^–1^ day^–1^), or an unspiked control feed (Ctr). We recorded different physiological parameters, determined Hg concentrations in brain tissue, and performed transcriptomic and proteomic analyses on hippocampus.

### Physiological parameters and mercury levels in the brain

**Fig. 1:**
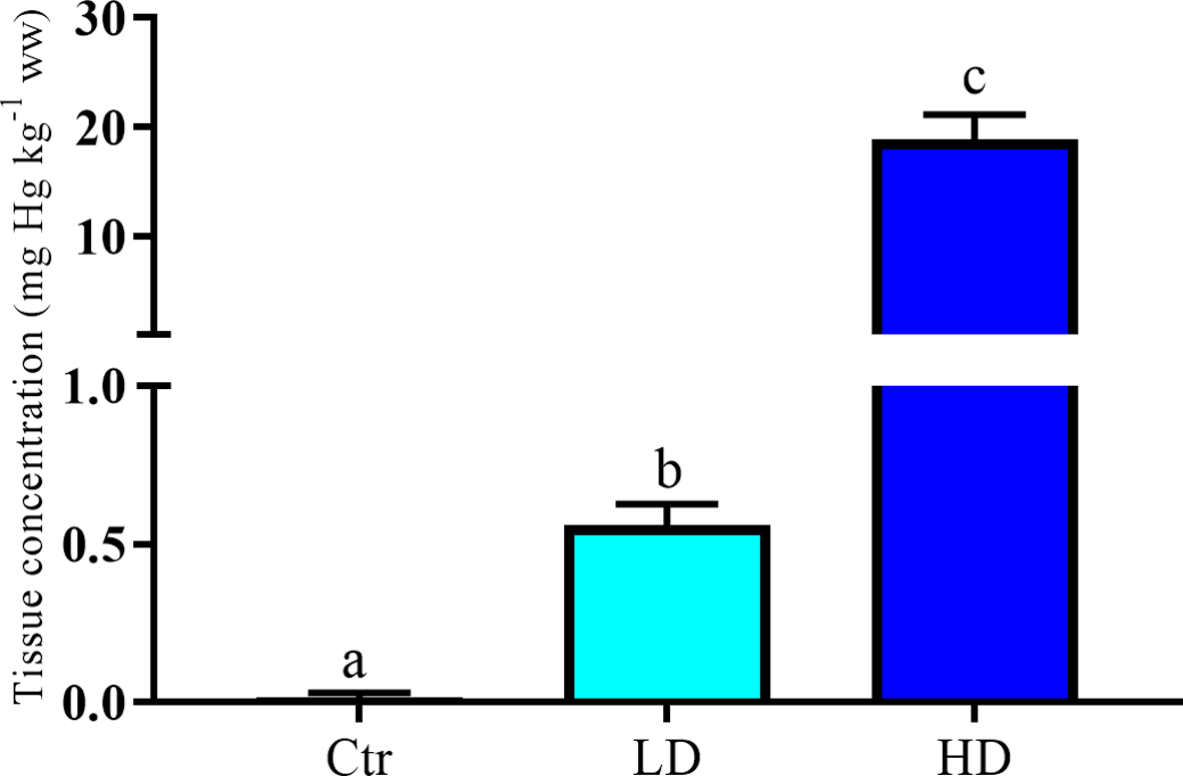
Hg concentration (mg Hg kg^–1^, ww) in cortex collected during the last week of the experiment. Data are presented as means with 95% confidence interval spread (*n* = 6). Different letters indicate statistical significance between groups. Statistics are performed using one-way ANOVA and Tukey's multiple comparison test on Box-Cox transformed data; raw data are presented in figure. Abbreviations: Ctr, control; LD, low dose; HD, high dose; ww, wet weight.

During the dietary exposure, no mortality or clinical signs of toxic MeHg effects were observed. Physiological endpoints, such as body weight, feed intake, body composition, and organ weights, and hematology of the mice were assessed throughout the trial and *post mortem* (Fig. S1 and Table S3). Apart from a small decrease in hemoglobin in the HD mice, no significant changes were observed between treatments for these parameters. As expected, the Hg levels in cortex were significantly (*P* < 0.05) higher in HD-fed mice than LD-fed mice and the Ctr (Fig. 1). Hg levels in LD-fed mice were also significantly (*P* < 0.05) higher than in the Ctr-fed mice.

### Principal component and hierarchical cluster analyses

**Fig. 2:**
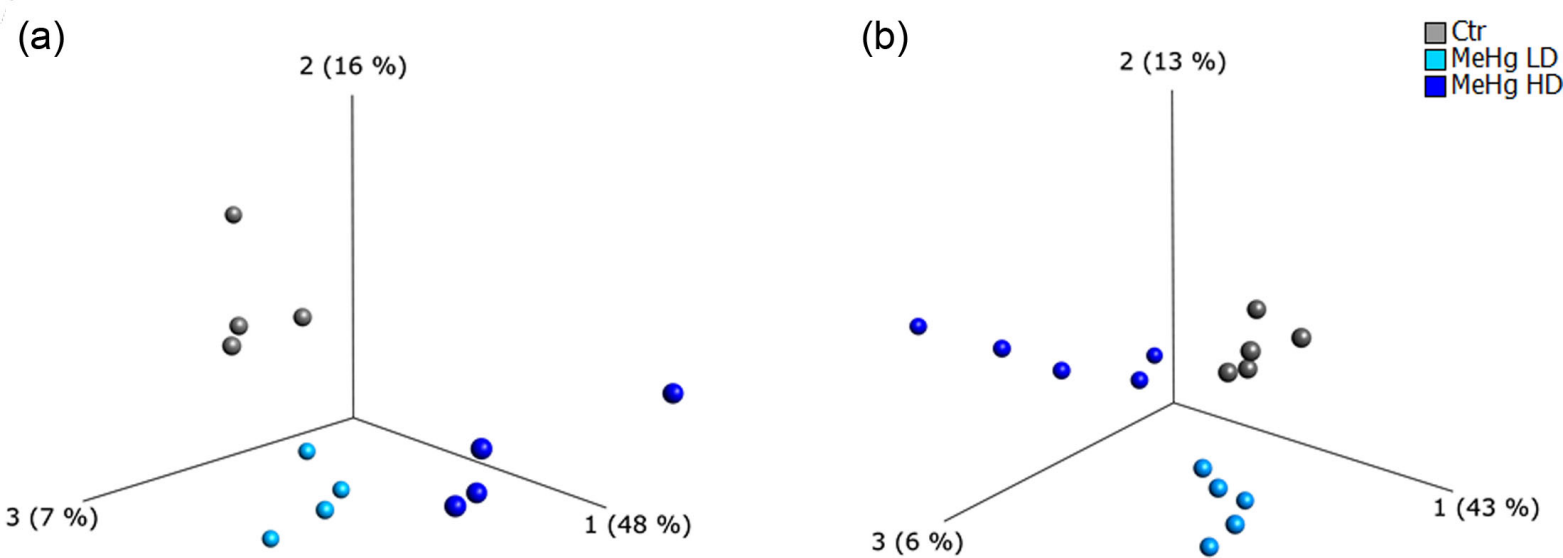
PCA of proteins (A, *n* = 4) and RNA transcripts (B, *n* = 5). Multigroup comparisons (ANOVA) were performed using QOE, *P* < 0.05. Abbreviations: Ctr, control; LD, low dose; HD, high dose.

A total of 2224 proteins and 21 412 RNA transcripts from hippocampus were analyzed using the Qlucore Omics Explorer (QOE, version 3.5, Qlucore, Lund, Sweden). PCA (Fig. 2) revealed a clear separation between the three groups (HD, LD, and Ctr) indicating distinct protein and RNA expression patterns. In total, 93 proteins and 1295 RNA transcripts were found to be significantly regulated (*P* < 0.05) following a one-way ANOVA analysis.

### Integration of transcriptomic and proteomic analyses, comparing low dose and high dose MeHg exposure

*Post hoc* analyses were performed for each treatment group following the one-way ANOVA. Comparing the LD group with the Ctr group revealed differential expression (*P* < 0.05) of 20 proteins (8 up/12 down) and 294 RNA transcripts (148 up/146 down). Compared with Ctr mice, 61 proteins (21 up/40 down) and 876 RNA transcripts (505 up/371 down) were differentially (*P* < 0.05) expressed in HD-fed mice.

**Fig. 3:**
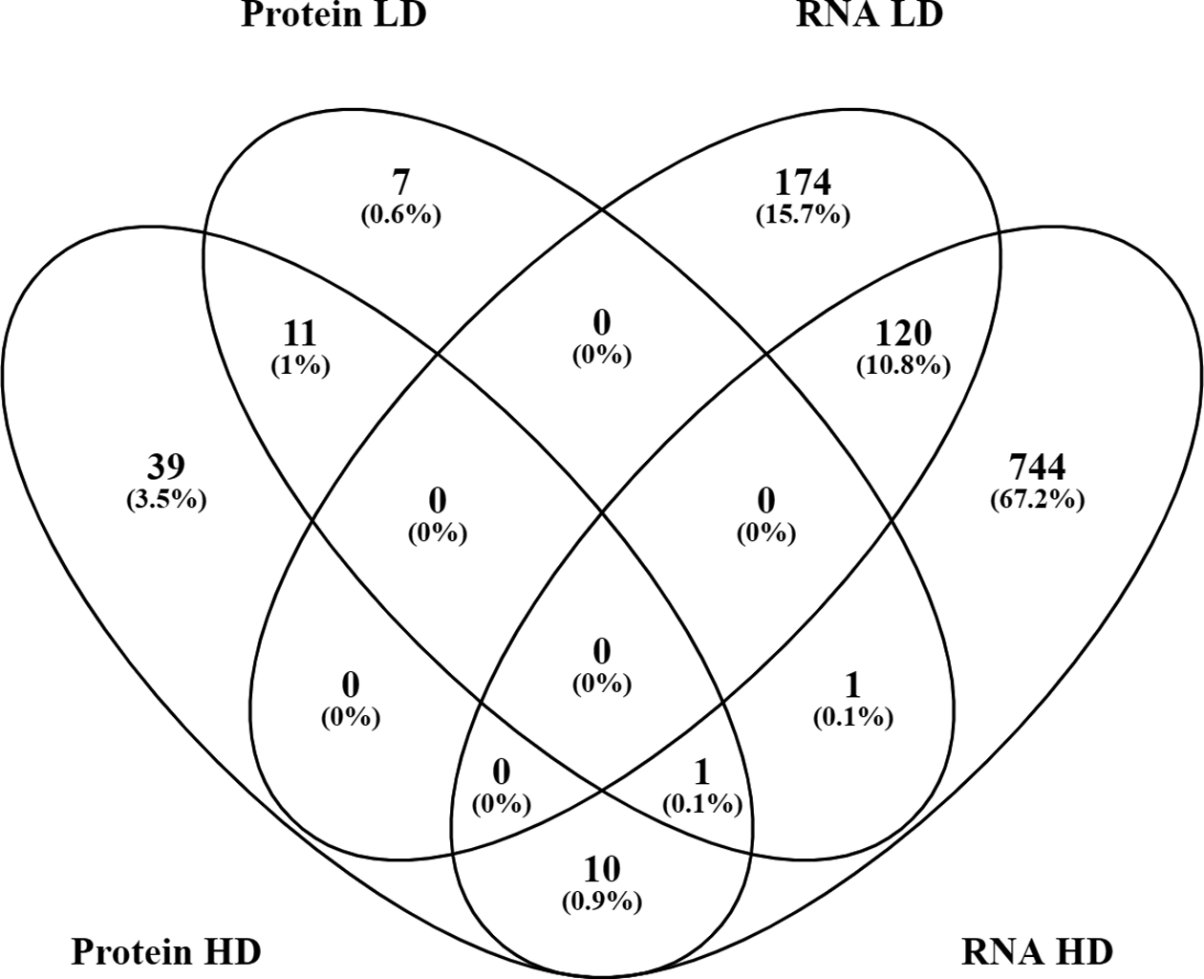
Overview of differentially expressed proteins and RNA overlapping between the two exposure groups. The differentially expressed features displayed are initially compared with the control group. Table of overlapping RNA and proteins is found in Table S5. Abbreviations: LD, low dose; HD, high dose; prot, proteins.

Venn analysis of differentially expressed features revealed overlapping expression patterns between the LD and HD groups both on protein and RNA levels (Fig. 3). In total, 120 of the differentially regulated RNA transcripts compared with Ctr were overlapping in the LD and HD groups (Table S5B). These overlapping transcripts are involved in a variety of molecular functions and processes, showing a top network in IPA related to hematological system development and function, inflammatory response, and tissue morphology (Table S5B). Of the differently regulated proteins in the LD and HD mice, 12 were overlapping, and showed a top network related to cell death and survival, cell morphology, and nervous system development and function (Table S5A).

The broad range of non-overlapping features and the consequent identification of several non-overlapping pathways in IPA between the LD and HD groups (Table 1) could indicate certain selective effects from a low and a high dietary MeHg exposure on protein and RNA expression in the hippocampus. These results will, however, be weaker statistically since these are only detected in single groups, without the confirmation of protein/RNA overlap. Protein expression in hippocampus reveals differences in affected molecular pathways in LD compared with HD exposed mice. The top four canonical pathways affected in the LD group are related to either modulation of the neurotransmitter glutamate or cytoskeletal dynamics, while all the top five canonical pathways regulated in the HD group are related to changes in metabolism (Table 1). However, based on RNA expression, most affected canonical pathways are related to immunological effects both for LD- and HD-fed mice. Albeit effects in the HD group were more pronounced in terms of significance and feature coverage, effects also spanned into vascular, immune, and xenobiotic metabolism (Table 1).

**Table 1.** Overview of main findings according to exposure group in the IPA software. Abbreviations: Ctr, control; LD, low dose; HD, high dose; RNA seq, RNA sequencing

Proteomic	*P*-value	No. of molecules	Proteomic	*P*-value	No. of molecules
LD			HD		
*Top canonical pathways*					
Glutamate-dependent acid resistance	0.0025	1	Glycerol degradation I	0.00014	2
Glutamate degradation III (via 4-aminobutyrate)	0.0062	1	Palmitate biosynthesis I	0.0076	1
RhoA signaling	0.0087	2	Glycerol-3-phosphate shuttle	0.0076	1
Signaling by Rho family GTPases	0.035	2	Fatty acid biosynthesis initiation II	0.0076	1
Mechanisms of viral exit from host cells	0.049	1	Tetrahydrobiopterin biosynthesis I	0.0114	1
*Top upstream regulators*					
NFIC	1.5E-06		MYRF	9.7E-10	
LEPR	2.2E-06		BCKDK	1.4E-08	
ST8SIA4	4.5E-06		EIF2AK4	1.4E-08	
BACE1	9.0E-06		TARDBP	2.2E-08	
FGFR2	1.5E-05		MTOR	4.3E-07	
					
RNA sequencing	*P*-value	No. of molecules	RNA sequencing	*P*-value	No. of molecules
LD			HD		
*Top canonical pathways*					
FXR/RXR activation	0.0129	4	Phagosome formation	1.4E-07	18
Systemic lupus erythematosus signaling	0.0219	5	Leptin signaling in obesity	3.2E-07	14
Nur77 signaling in T lymphocytes	0.0222	3	Adrenomedullin signaling pathway	1.9E-06	22
TREM1 signaling	0.0243	3	Dendritic cell maturation	2.5E-06	19
Cholecystokinin/gastrin-mediated signaling	0.0274	3	Xenobiotic metabolism signaling	6.7E-06	25
*Top upstream regulators*					
AGER	0.00162		KDM1A	2.5E-21	
IL12B	0.00226		MAPT	1.2E-16	
PTGER2	0.00495		IL10	2.1E-11	
B4GALNT1	0.00575		ST8SIA1	4.3E-10	
SSB	0.00931		B4GALNT1	9.7E-10	

### Joint findings between RNA transcriptomic and proteomic in hippocampus

**Fig. 4:**
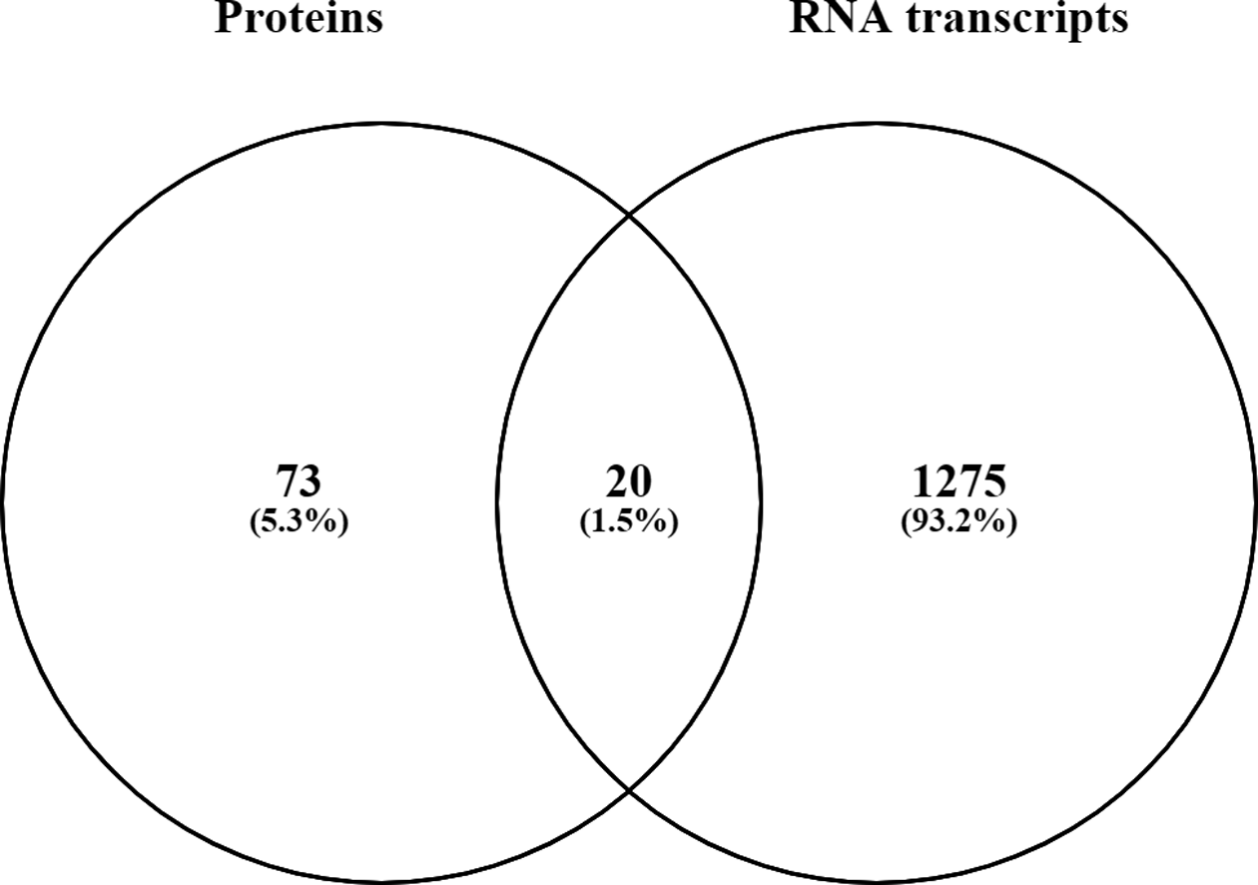
Comparison of differentially expressed proteins and RNA by MeHg exposure (LD and HD related to Ctr) detected through proteomics and RNA sequencing, respectively (one-way ANOVA, *P* < 0.05). Features are matched and overlapped independent of MeHg exposure dose. The overlapping features are presented in Table 2.

To investigate overlapping proteins and RNA transcripts differently expressed after MeHg exposure, all features affected by MeHg exposure independent of dose were visualized in a Venn diagram (Fig. 4).

A total of 20 features were significantly (*P* < 0.05, ANOVA) regulated both at the RNA and protein levels (Table 2). Of these, 12 specific features were significantly regulated (*P* < 0.05) following a two-group planned comparison *post hoc* test both at RNA expression and protein abundance levels. These 12 overlapping features were apolipoprotein E (*APOE*), dual specificity protein phosphatase 3 (DUS3), glial fibrillary acidic protein (GFAP), annexin A7 (ANXA7), 2-iminobutanoate/2-iminopropanoate deaminase (*RIDA*), catalase (*CATA)*, tetraspanin-2 (TSN2), ornithine aminotransferase, mitochondrial (*OAT*), vesicle-associated membrane protein 1 (*VAMP1*), complement C1q subcomponent subunit B (*C1QB*), GTP-binding protein Di-Ras2 (*DIRA2*), and glutamate decarboxylase 1 (DCE1), all mainly affected by the high dose MeHg exposure. Additionally, GFAP and DCE1 were affected by the low dose MeHg on protein level. Of the abovementioned features, seven were upregulated and five were downregulated. Features upregulated by MeHg exposure were involved in antioxidant activity and handling of toxic metabolites (*APOE, CATA, RIDA*), calcium regulation (ANXA7), the neurological function (GFAP), the immune system (*C1QB*), and amino acid synthesis (*OAT*). Of the downregulated features, one was involved in GTPase activity and neuronal development (*DIRA2*), downregulation of MAP kinases and subsequent effects on cellular proliferation and differentiation (DUS3), vesicle transport (*VAMP1*), regulation of neurotransmitter GABA (DCE1), and oligodendrocyte differentiation (*TSN2*).

**Table 2.** Features significantly overlapping between proteomic and RNA sequencing in main one-way ANOVA (*P* < 0.05) independent of MeHg exposure dose (Fig. 5). Yellow indicates upregulation and blue downregulation when compared with Ctr in two-group planned comparison test. Abbreviations: Ctr, control; LD, low dose; HD, high dose.

				Proteomics					RNA transcripts				
Ensembl ID	UniProt ID	Abb.	Prot. name	ANOVA (*P*)	Ctr vs LD	FC	Ctr vs HD	FC	ANOVA (*P*)	Ctr vs LD	FC	Ctr vs HD	FC
ENSMUSG00000002985	P08226	APOE	Apolipoprotein E	0.001	0.096	0.87	0.007	1.52	0.015	0.566	1.05	0.013	1.40
ENSMUSG00000003518	Q9D7X3	DUS3	Dual specificity protein phosphatase 3	0.009	0.802	0.99	0.009	0.82	0.037	0.641	0.96	0.011	0.79
ENSMUSG00000003585	Q99J08	S14L2	SEC14-like protein 2	0.033	0.614	0.96	0.081	1.19	0.041	0.057	1.17	0.037	1.19
ENSMUSG00000007891	P18242	CATD	Cathepsin D	0.023	0.299	0.93	0.066	1.48	0.003	0.690	1.03	0.011	1.94
ENSMUSG00000015085	O55026	ENTP2	Ectonucleoside triphosphate diphosphohydrolase 2	0.033	0.535	0.93	0.056	1.27	0.012	0.088	0.81	0.133	1.26
ENSMUSG00000020932	P03995	GFAP	Glial fibrillary acidic protein (GFAP)	0.001	0.024	0.82	0.007	2.97	0.000	0.356	1.08	0.002	5.24
ENSMUSG00000021814	Q07076	ANXA7	Annexin A7	0.007	0.093	0.91	0.041	1.14	0.008	0.364	0.96	0.043	1.12
ENSMUSG00000021939	P10605	CATB	Cathepsin B	0.015	0.558	0.95	0.044	1.39	0.048	0.632	0.98	0.088	1.27
ENSMUSG00000022323	P52760	RIDA	2-Iminobutanoate/2-iminopropanoate deaminase	0.003	0.937	1.01	0.006	1.48	0.000	0.790	1.02	0.000	1.53
ENSMUSG00000027187	P24270	CATA	Catalase	0.019	0.052	2.01	0.021	2.17	0.005	0.264	1.03	0.013	1.22
ENSMUSG00000027712	P48036	ANXA5	Annexin A5	0.017	0.170	0.92	0.079	1.14	0.033	0.382	0.93	0.089	1.39
ENSMUSG00000027858	Q922J6	TSN2	Tetraspanin-2 (Tspan-2)	0.045	0.214	0.76	0.025	0.58	0.015	0.962	1.00	0.012	0.82
ENSMUSG00000030337	Q62442	VAMP1	Vesicle-associated membrane protein 1 (VAMP-1) (Synaptobrevin-1)	0.022	0.962	0.99	0.009	0.63	0.001	0.979	1.00	0.003	0.71
ENSMUSG00000030934	P29758	OAT	Ornithine aminotransferase, mitochondrial	0.006	0.796	1.03	0.004	1.39	0.003	0.706	0.99	0.007	1.16
ENSMUSG00000036905	P14106	C1QB	Complement C1q subcomponent subunit B	0.008	0.880	1.08	0.008	5.10	0.002	0.195	1.29	0.003	3.29
ENSMUSG00000047557	P70202	LXN	Latexin	0.048	0.350	0.95	0.118	1.16	0.001	0.927	1.01	0.004	1.52
ENSMUSG00000047842	Q5PR73	DIRA2	GTP-binding protein Di-Ras2	0.020	0.249	0.89	0.009	0.75	0.025	0.619	0.98	0.039	0.82
ENSMUSG00000057193	Q8BY89	CTL2	Choline transporter-like protein 2	0.018	0.397	0.89	0.033	0.40	0.049	0.225	0.95	0.126	1.13
ENSMUSG00000059447	Q99JY0	ECHB	Trifunctional enzyme subunit beta, mitochondrial	0.042	0.315	0.93	0.090	1.17	0.010	0.540	0.98	0.027	1.10
ENSMUSG00000070880	P48318	DCE1	Glutamate decarboxylase 1	0.005	0.031	1.12	0.075	0.86	0.001	0.097	0.89	0.000	0.73

**Fig. 5:**
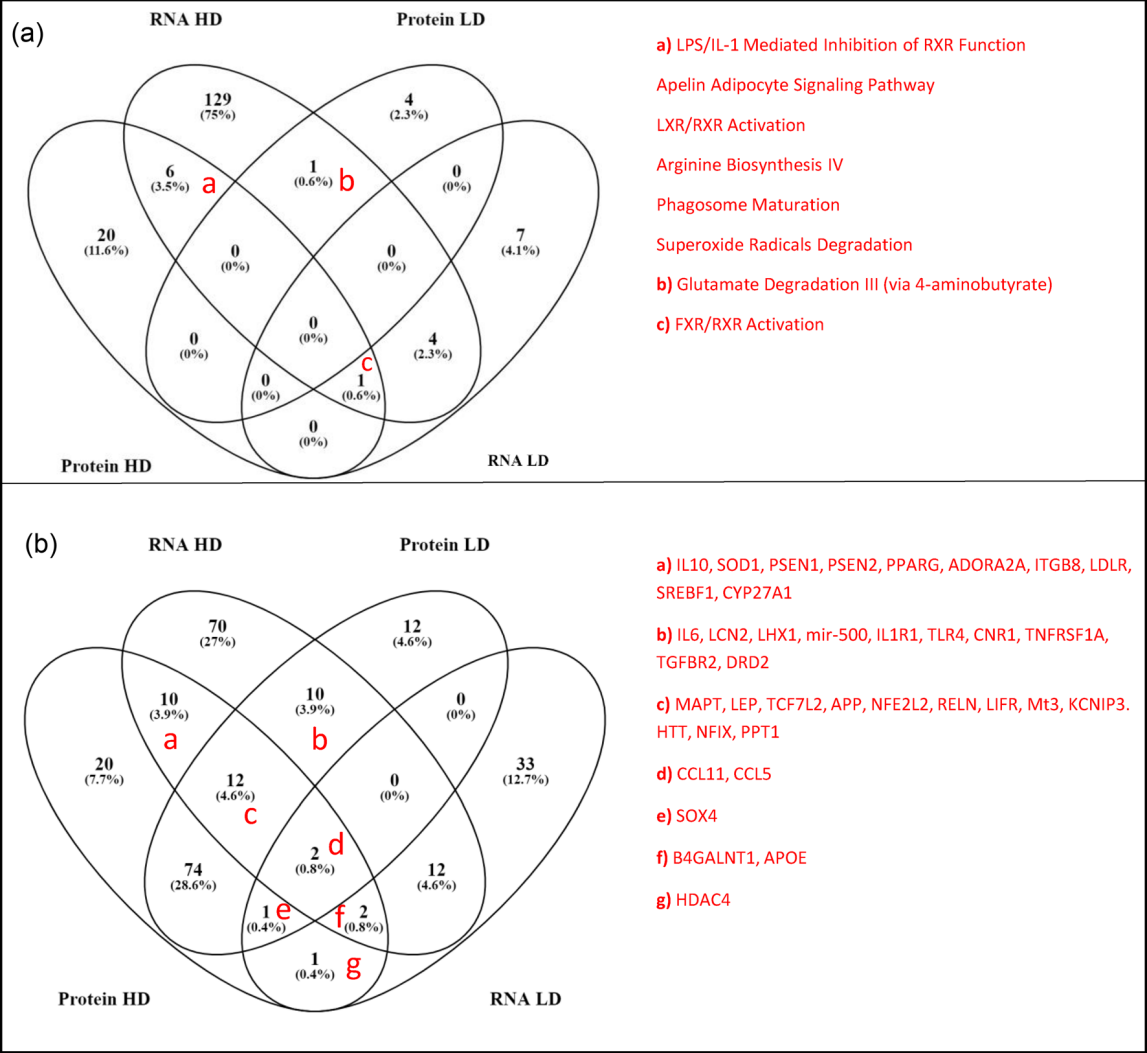
Overlapping canonical pathways and upstream regulators between RNA sequencing and proteomics, and LD and HD. (a) Overlapping significant canonical pathways (*P* < 0.05); “a” denotes overlap between HD RNA and HD protein; “b” denotes overlap for LD protein and HD RNA; and “c” denotes overlap for HD both for RNA and proteins and LD RNA. (b) Overlapping significant upstream regulators (*P* < 0.05); “a” denotes overlap between HD RNA and HD protein; “b” denotes overlap for LD protein and HD RNA; “c” denotes overlap for HD both for RNA and proteins and LD protein; “d” denotes overlap for all groups; “e” denotes overlap for LD and HD for proteins and LD for RNA; “f” denotes overlap for LD and HD for RNA and HD protein; and “g” denotes overlap for HD protein and LD RNA.

To further investigate the MeHg-induced effects, canonical pathways and upstream regulators, which were predicted to be significantly affected (*P* < 0.05) by IPA (Table S4A and B) for both RNA transcripts and protein differential expression, were determined (Fig. 5). Identified canonical pathways were lipopolysaccharide/interleukin-1 (LPS/IL-1) mediated inhibition of RXR function, apelin adipocyte signaling pathway, LXR/RXR activation, arginine biosynthesis IV, phagosome maturation, FXR/RXR activation, superoxide radical degradation, and glutamate degradation III. Further, several suggested upstream regulators were also found to be affected by both RNA and protein analyses dependent on dose (Fig. 5b).

## Discussion

In this study, using transcriptomic and proteomic analyses of hippocampus from BALB/c mice, we demonstrate that known effects of MeHg, such as differentially expressed genes and proteins indicative of inflammation, metabolism, and neurotoxicity, are present at both low dose of MeHg (0.28 mg Hg kg^–1^ feed) and high dose of MeHg exposure (5 mg Hg kg^–1^ feed). The combined findings from both proteomic and RNA sequencing increase the confidence in the validity of these results irrespective of the statistical challenges encountered when both approaches were applied independently.

Seafood is the main dietary exposure source for MeHg in humans.^6^ Several fish species contain levels comparable to, or above, the levels of MeHg in the low dose diets of the present trial.^11,59^ Bioaccumulation of MeHg along the aquatic food chain can lead to higher levels of mercury in predatory fish and marine mammals.^2,5,60^ This fact presents a 2-fold threat. First, contents in seafood and seafood products consumed by humans may contain very high levels of MeHg.^61–^^64^ Second, in an ecotoxicological perspective, MeHg can pose a risk to the wildlife itself.^65^ Total mercury levels assessed in brain of several wildlife species like mink, striped dolphins, and otters have been detected in the same order of magnitude as observed in the mice exposed to the high dose MeHg in the present trial.^22^,^66–^^68^ Levels of Hg detected in the brain of the mice exposed to both LD and HD are also comparable to levels predicted in humans living in contaminated areas from studies using hair measurements.^69,70^ Previous studies have determined neurophysiological alterations by prenatal exposure to low levels of MeHg^71^; these authors have therefore stated that there are no “safe levels” of MeHg, which underlines the importance of low dose studies of MeHg.

No apparent changes in gross pathology were observed in our study. Still, alteration in the proteome and transcriptome, indicating toxic effects in the hippocampus after MeHg exposure, was demonstrated. Results from previous animal trials using similar low doses as the present study have shown motor function damage, coordination deficits, and learning impairments in rats.^72^ Also, reduced neuronal cell density and increased oxidative stress together with decreased antioxidant capacity have been observed in rats exposed to similar low doses of MeHg.^35,73^ Comparable levels of MeHg in cortex, as demonstrated in our study, have previously been reported to reduce cognitive performance.^74^ Other proteomic studies have in line with our findings displayed several effects of MeHg on molecular pathways, which include neurodegenerative processes and altered energy metabolism,^34,35^ indicating that despite the lack of pathology, early indicators of adverse effects by MeHg can still be determined in the hippocampal proteome and transcriptome.

A clear separation of the expression patterns in LD mice from both Ctr and HD mice was observed, suggesting that high and low MeHg doses can act by different mechanisms. However, due to relatively low statistical power, these results could also potentially be a statistical artifact due to too low sample size to determine sufficient effect size in the LD group. Still, together with the notion that MeHg can exert a non-monotonic effect,^18,19^ our results indicate the importance of investigating the effects of MeHg exposure at varying levels. Although clear differences between doses were found, we also detected some consistent effects across exposure levels, such as effects on energy metabolism and neurological development. This is in accordance with previous reports on MeHg toxicity in which effects on neuronal development and energy metabolism were detected.^4,34,35,75^ Effects of low levels of MeHg exposure have been reported in humans, resulting in neurocognitive effects through prenatal exposure,^76^ and the general acknowledgement of MeHg as a neurotoxic compound has been firmly established.^27^ Our results are in accordance with earlier studies demonstrating that a range of MeHg effects are dose dependent, but also according to our findings, transcriptomic and proteomic signatures indicate that potentially adverse neurological implications of early-life MeHg exposure can be present already at low dose exposure levels, whereas a higher dose may be required to observe the fuller extent of molecular pathways affected by MeHg.

Furthermore, when assessing overlapping canonical pathways affected by both low and high dose MeHg, we found that both LPS/IL-1 mediated inhibition of RXR function and superoxide radical degradation were significantly affected by both low dose and high dose MeHg on protein level. The RXRs play an important regulatory role in metabolism, such as glucose, fatty acid, and cholesterol metabolism,^77^ corroborating the notion that metabolism is affected independent of dose. Superoxide radical degradation is mainly mediated by the feature *CATA*, which was found to be upregulated both after low dose and high dose exposure on the protein level and after high dose exposure on the RNA level. Superoxide dismutase (SOD) transcripts were also regulated in the high dose. Both CATA and SOD are important antioxidants important for maintenance of oxidative homeostasis in the cells.^78,79^ Oxidative stress is a well-known molecular mechanism of MeHg,^27^ and is in our study affected independently of dose.

An important aspect of this study is the combined use of RNA sequencing and proteomic profiling. Of note, the overlapping features identified at both RNA and protein levels were mostly concomitantly up- or downregulated suggesting consistency in regulation between the two methods. It should be noted that brain proteomics does not necessarily correspond well to gene expression^42^ due to the substantial role of post-transcriptional, translational, and protein degradation in the brain.^80^ The latter may partly explain the low correlation between RNA-seq and proteomic findings in our study. Furthermore, post-translational modifications have also been highlighted as important for higher brain functions,^81^ and these have not been assessed in this study. Still, effects conserved between RNA and protein may be strong indications of a MeHg-induced response, despite general low effect sizes of differentially regulated proteins.

Most corresponding findings between RNA and proteins were in the high dose-exposed mice, which could indicate that expression of proteins and RNA transcripts in the brain is highly regulated until a certain threshold dose occurs. Expression levels of *VAMP1, DIRA2*, and *TSN2* involved in exocytosis and the release of neurotransmitter, glutamatergic and catecholaminergic neurons and neurogenesis, and oligodendrocyte development and immune functions, respectively,^82–^^86^ were downregulated indicating neurotoxicity as previously observed.^87,88^ Additionally, expression levels of *APOE, CATA*, and *RIDA* involved in antioxidative defense and handling of toxic reactive metabolites were upregulated, and superoxide radical degradation was observed as a canonical pathway in both transcriptomic and proteomic analyses. Oxidative stress has been suggested as a driving force in MeHg-induced neurotoxicity,^27,89^ where downregulation of *VAMP1, DIRA2*, and *TSN2* may indicate a detrimental effect of MeHg on the neuronal cells and neurotransmitter function. In addition, the possible increase in reactive oxygen species and oxidative stress by MeHg may have led to a compensatory upregulation of the *APOE, RIDA*, and *CATA* features with antioxidative properties. The additional upregulation of the protein *CATA* in the low dose-exposed mice may suggest that this antioxidant can be an early biomarker of MeHg toxicity, supporting oxidative stress as an effect of MeHg evident already by low dose exposures. However, whether the expression levels of specific features are a direct cause of MeHg exposure or compensatory mechanisms cannot firmly be established emphasizing the complexity of omics interpretation.

### Conclusion

We have shown that MeHg can induce differential protein and RNA expression at doses corresponding to relevant human and wildlife exposure levels. We identified dose-independent MeHg-regulated signatures involving effects on energy metabolism, oxidative homeostasis, and the nervous system function. Further, we have shown indications that expression of different proteins and RNA transcripts as well as associated pathways can be affected differently by low and high dose MeHg exposure, emphasizing the importance of dose-response studies when examining the toxicological effects of MeHg.

## Supplementary Material

mfab022_Supplemental_Tables

## Data Availability

Protein and RNA expression data are provided in Tables S1 and S2, respectively. RNA raw data have been uploaded to the gene expression omnibus (GEO) with accession number GSE135381.
